# Supplementary material for: Chemistry-driven changes strongly influence climate forcing from vegetation emissions
Source: Nat Commun. 2022 Nov 23;13:7202. doi: 10.1038/s41467-022-34944-9 (PMC9684129; doi:10.1038/s41467-022-34944-9)
Supplement: Supplementary file 2 — Supplementary information [file 41467_2022_34944_MOESM2_ESM.pdf]

Supplementary Information

James Weber, Scott Archer-Nicholls, N. Luke Abraham, Youngsub Matthew Shin, Paul Griffiths, Daniel P. Grosvenor, Catherine E. Scott and Alex T. Archibald

Corresponding author: James Weber
Email: j.weber@sheffield.ac.uk

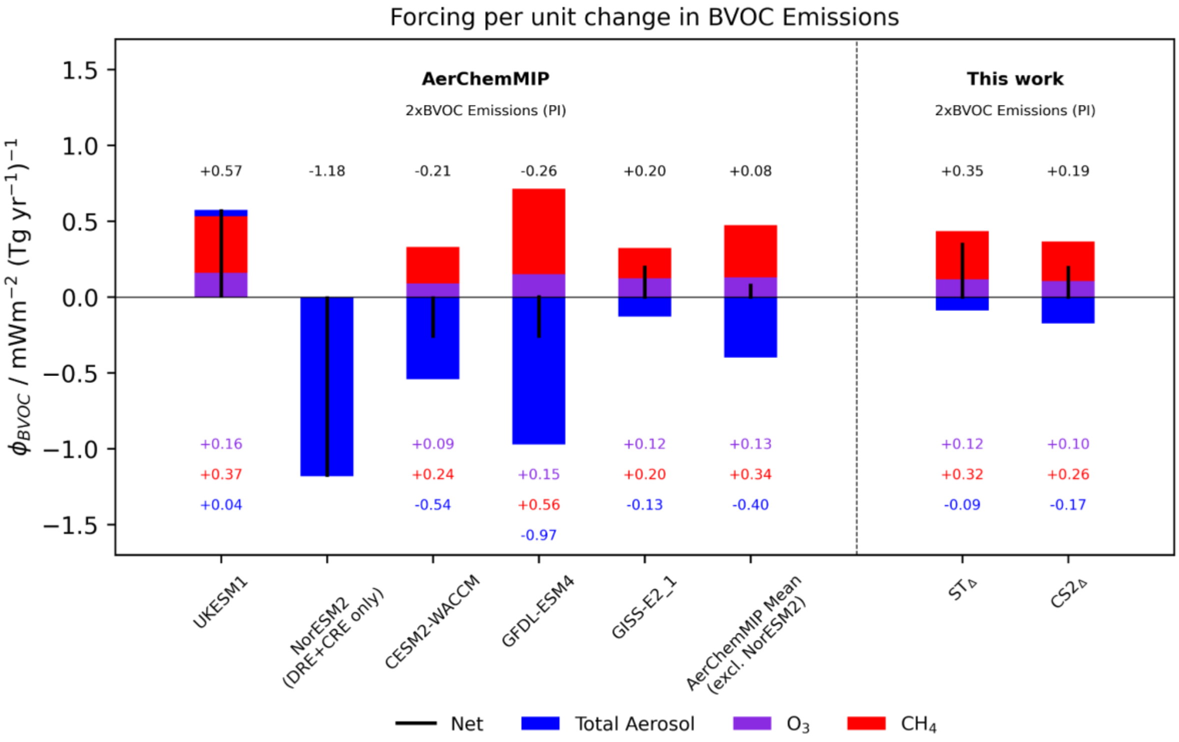

Fig. S1. Forcing per unit change in BVOC emissions from the AerChemMIP 2xBVOC study (Thornhill et al., 2021) and this work.

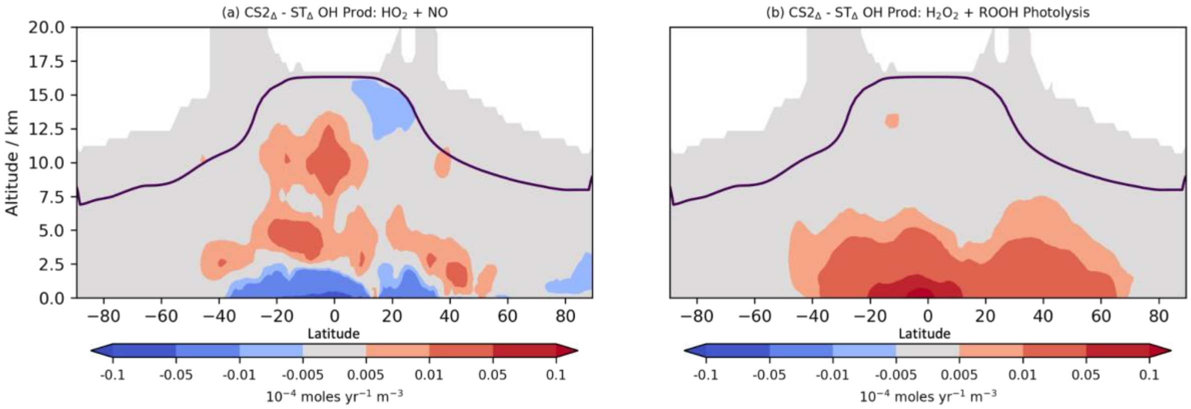

Fig. S2. OH production differences. Zonal mean difference between Strat-Trop (ST_Δ) and CRI-Strat 2 ($CS2_\Delta$) for OH production from (a) HO_2 + NO and (b) H_2O_2 and other peroxides (ROOH).

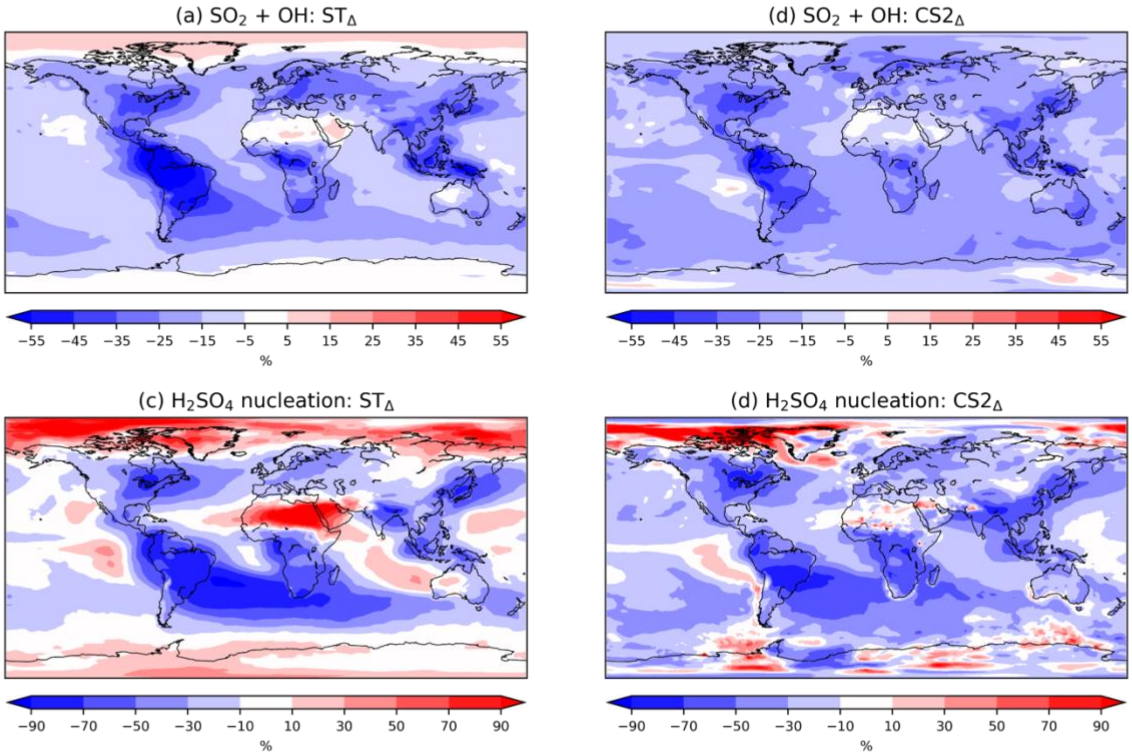

Fig. S3. SO_2 oxidation and H_2SO_4 nucleation. Percentage change in vertically integrated flux of SO_2 + OH for (a) Strat-Trop (ST_Δ) and (b) CRI-Strat 2 ($CS2_\Delta$) and nucleation flux of new particle formation from H_2SO_4 for (c) ST_Δ and (d) $CS2_\Delta$.

Supplementary References

1. G. Thornhill et al. Climate-driven chemistry and aerosol feedbacks in CMIP6 Earth system models. *Atmospheric Chemistry and Physics* 21.2, 1105-1126 (2021).
